# Blast Crisis

**DOI:** 10.21980/J8W35K

**Published:** 2020-04-15

**Authors:** Margaret Kirwin, Jennifer Yee

**Affiliations:** * The Ohio State University, Department of Emergency Medicine, Columbus OH

## Abstract

**Audience:**

This scenario was developed to educate emergency medicine residents on the diagnosis and management of blast crisis.

**Introduction:**

Chronic myeloid leukemia (CML) makes up 15% of diagnosed adult leukemias with the median age of diagnosis being 67 years old. Chronic myeloid leukemia consists of three phases: chronic, accelerated, and blast phases. Most patients are initially diagnosed while in the chronic phase.^1^ Of those diagnosed in the chronic phase and being treated with a tyrosine kinase inhibitor (TKI), about 1% –1.5% of CML patients per year will subsequently transform into an advanced phase or blast crisis.^2^ While rare, blast crisis is considered an oncologic emergency, with increased mortality occurring primarily from subsequent infections or bleeding. Therefore, emergency physicians must be familiar with its clinical presentation and subsequent management.

**Educational Objectives:**

By the end of this simulation, the participant will be able to: 1) create a thorough differential for the undifferentiated febrile, altered patient, 2) identify the signs and symptoms of blast crisis, 3) describe proper resuscitation of a patient in blast crisis, and 4) describe the indications, steps, and contraindications of performing a lumbar puncture.

**Educational Methods:**

This session was conducted using high-fidelity simulation, followed by a debriefing session and lecture on the diagnosis, differential diagnosis, and management of blast crisis. Debriefing methods may be left to the discretion of participants, but the authors have used advocacy-inquiry techniques. This scenario may also be run as an oral boards case.

**Research Methods:**

Our simulation center’s feedback form is based on the Center of Medical Simulation’s Debriefing Assessment for Simulation in Healthcare (DASH) Student Version Short Form, with the inclusion of required qualitative feedback if an element was scored less than a 6 or 7.

**Results:**

This session received all 6 or 7 scores (consistently effective/very good or extremely effective/outstanding). During the debriefing session, feedback from the residents was largely positive. They appreciated reviewing the broad differential of altered mental status and oncologic emergencies. While many groups anchored on the diagnosis of encephalitis, they also expressed that after this experience, blast crisis would be added to their differential for patients with CML.

**Discussion:**

This is a cost-effective method for reviewing blast crisis. Learners were able to identify more common causes of altered mental status in their differentials, but without further prompting, they were unable to ultimately come up with the diagnosis of blast crisis. Our main take-away is to continue reviewing oncologic emergencies as a part of our residency curriculum.

**Topics:**

Medical simulation, chronic myeloid leukemia, blast crisis, leukostasis, emergency medicine, oncologic emergencies, hematologic emergencies.

## USER GUIDE

[Table t1-jetem-5-2-s55]

**Table t1-jetem-5-2-s55:** 

List of Resources:	
Abstract	55
User Guide	57
Instructor Materials	59
Operator Materials	69
Debriefing and Evaluation Pearls	70
Simulation Assessment	73

**Learner Audience:**

Interns, junior residents, senior residents

**Time Required for Implementation:**

Instructor Preparation: 30 minutes

Time for case: 20 minutes

Time for debriefing: 40 minutes

**Recommended Number of Learners per Instructor:**

4

**Topics:**

Medical simulation, chronic myeloid leukemia, blast crisis, leukostasis, emergency medicine, oncologic emergencies, hematologic emergencies.

**Objectives:**

By the end of this simulation session, the learner will be able to:

1. Create a thorough differential for the undifferentiated febrile, altered patient.
2. Identify the signs and symptoms of blast crisis.
3. Describe proper resuscitation of a patient in blast crisis.
4. Describe the indications, steps, and contraindications of performing a lumbar puncture.

### Linked objectives and methods

Blast crisis is still a major challenge in the management of CML, although its incidence has greatly decreased. With the development of TKIs, the rate of progression to blast crisis has reduced to 1% to 1.5% per year.^2^ Blast crisis is life-threatening and requires urgent management. It is important that emergency physicians are able to recognize blast crisis and promptly initiate treatment. This simulation scenario allows learners to review the differential of the altered patient (objective 1). Learners will ultimately need to identity the signs and symptoms of blast crisis (objective 2), practice mobilizing the appropriate resources, and begin emergent resuscitation (objective 3). The learners will also need to identify possible contraindications to certain procedures such as lumbar puncture, which would usually be included in the work up of this patient (objective 4). Afterwards, there will be a debriefing session to discuss the differential, pathophysiology and management of blast crisis (objectives 1–3).

### Recommended pre-reading for instructor

We recommend that instructors review literature regarding blast crisis, including epidemiology, presenting signs/symptoms, diagnosis, and management. High-yield readings include the following:

- Porcu P, Cripe LD, Ng EW, et al. Hyperleukocytic leukemias and leukostasis: a review of pathophysiology, clinical presentation and management. *Leuk Lymphoma.* 2000;39(1–2):1–18. doi:10.3109/10428190009053534.
- Saußele S, Silver RT. Management of chronic myeloid leukemia in blast crisis. *Ann Hematol*. 2015;94, Suppl 2: S159–165. doi:10.1007/s00277-015-2324-0.

### Results and tips for successful implementation

This simulation scenario was conducted approximately three times for a total of eight emergency medicine residents. We found that the residents struggled to make the diagnosis. While other groups entertained the potential for encephalitis, all three groups were unable to come up with the diagnosis even when provided the CBC and pertinent history. Some groups were able to identify that lumbar puncture would likely be contraindicated due to the patient’s thrombocytopenia. During the debriefing, it was discovered that blast crisis was considered by some individuals in two of the groups’ differentials but was ultimately not voiced to the rest of the group. The residents were able to come up with other oncologic emergencies in their differentials such as tumor lysis syndrome, but they were less familiar with blast crisis and the appropriate management.

Our simulation center’s feedback form is based on the Center of Medical Simulation’s Debriefing Assessment for Simulation in Healthcare (DASH) Student Version Short Form with the inclusion of required qualitative feedback if an element was scored less than a 6 or 7. This session received all 6 or 7 scores (consistently effective/very good or extremely effective/outstanding). Mean scores are as follows: Category 1 (the instructor set the stage for an engaging learning experience) 6.125, Category 2 (the instructor maintained an engaging context for learning) 6.375, Category 3 (the instructor structured the debriefing in an organized way) 6.5, Category 4 (the instructor provoked in-depth discussions that led me to reflect on my performance) 6.25, Category 5 (the instructor identified what I did well or poorly – and why) 6.125, and Category 6 (the instructor helped me see how to improve or how to sustain good performance) 6.25. Our form also includes an area for general feedback about the case at the end. Comments included “Good case; would have liked patient to have hard signs of end organ damage such as stroke or myocardial infarction. Would have made push for leukapheresis/cytoreduction easier.”

The scenario was not assessed with pre-testing. This simulation was written to be performed as a high-fidelity simulation scenario but may also be used as a mock oral board case. No modifications are suggested to modify to a low-fidelity or mock oral boards case.

## Supplementary Information



## Figures and Tables

**Table t2-jetem-5-2-s55:** 

#1	Complete blood count (CBC) with differential
#2	Peripheral smear
#3	Basic metabolic panel (BMP)
#4	Hepatic function panel
#5	Coagulation factors
#6	Lactate dehydrogenase
#7	Uric acid
#8	Urinalysis
#9	Rapid influenza testing
#10	Electrocardiogram (ECG)
#11	Computed tomography (CT) head
#12	Chest X-ray (CXR)

**Table t3-jetem-5-2-s55:** 

**Vitals:**	Heart Rate (HR) 107	Blood Pressure (BP) 140/84	Respiratory Rate (RR) 20
	Temperature (T) 101.3°F	Oxygen Saturation (O_2_Sat) 100% on room air	
	Weight (Wt) 70 kg		

**Table t4-jetem-5-2-s55:** 

*Complete blood count (CBC)*	
White blood count (WBC)	102.3 ×1000/mm^3^
Hemoglobin (Hgb)	8.2 g/dL
Hematocrit (HCT)	25.6%
Platelet (Plt)	55 ×1000/mm^3^
Neutrophils	43%
Lymphocytes	23%
Monocytes	8%
Eosinophils	2%
Basophils	24%

**Figure f1-jetem-5-2-s55:**
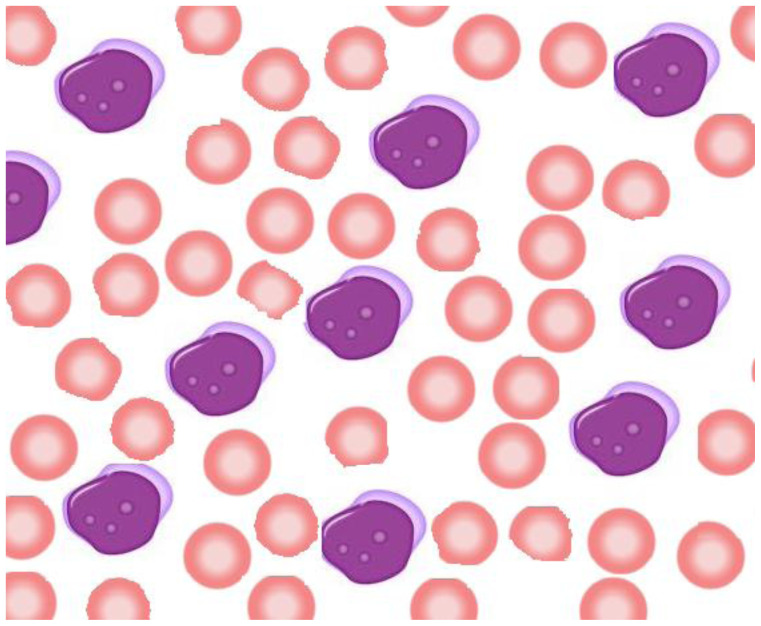


**Table t5-jetem-5-2-s55:** 

*Basic metabolic panel (BMP)*	
Sodium	138 mEq/L
Chloride	105 mEq/L
Potassium	3.9 mEq/L
Bicarbonate (HCO_3_)	27 mEq/L
Blood Urea Nitrogen (BUN)	32 mg/dL
Creatine (Cr)	1.4 mg/dL
Glucose	119 mg/dL
Calcium	9.5 mg/dL

**Table t6-jetem-5-2-s55:** 

*Coagulation panel*	
Prothrombin Time (PT)	11.2 seconds
International Normalized Ratio (INR)	1.0
Partial Thromboplastin Time (PTT)	31 seconds

**Table t7-jetem-5-2-s55:** 

*Lactate dehydrogenase*	460 units/L

**Table t8-jetem-5-2-s55:** 

*Uric Acid*	8.1 mg/dL

**Table t9-jetem-5-2-s55:** 

*Urinalysis (UA)*	
Leukocyte esterase	negative
Nitrites	negative
Blood	none
Protein	none
Ketones	1+
Glucose	none
Color	dark yellow
White blood cells (WBC)	0–5 WBCs/high powered field (HPF)
Red blood cells (RBC)	0–5 RBCs/HPF
Squamous epithelial cells	0–5 cells/HPF
Specific gravity	1.015

**Table t10-jetem-5-2-s55:** 

*Rapid influenza testing*
negative

**Figure f2-jetem-5-2-s55:**
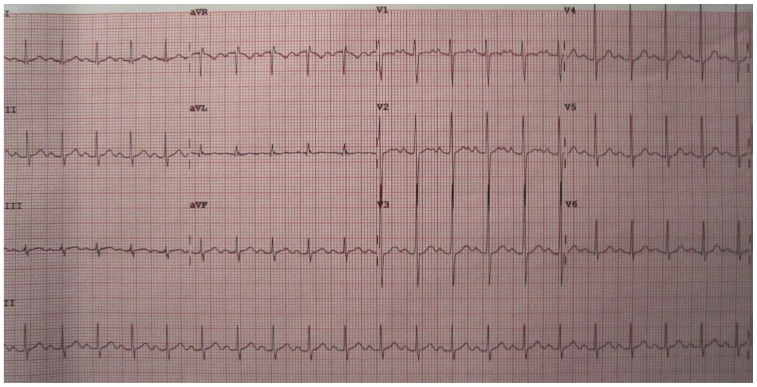


**Figure f3-jetem-5-2-s55:**
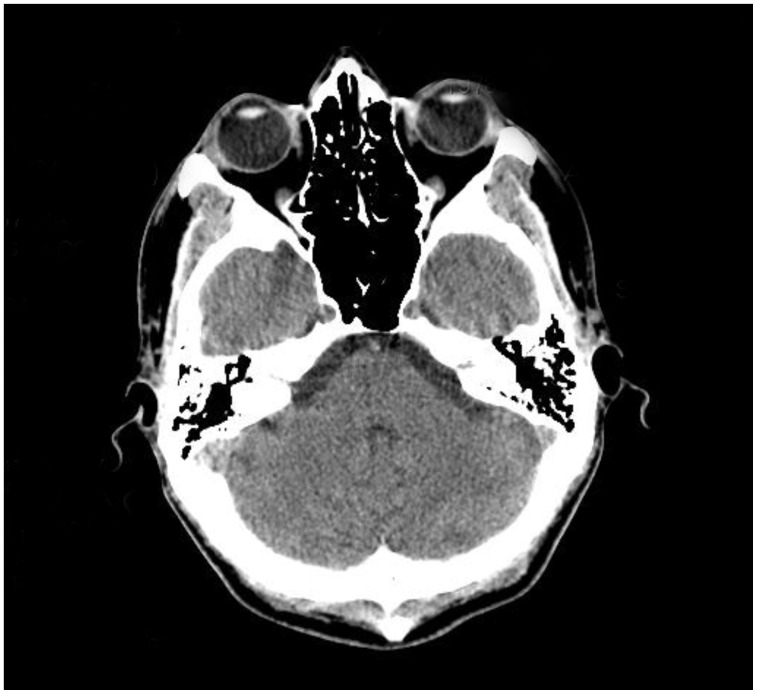


**Figure f4-jetem-5-2-s55:**
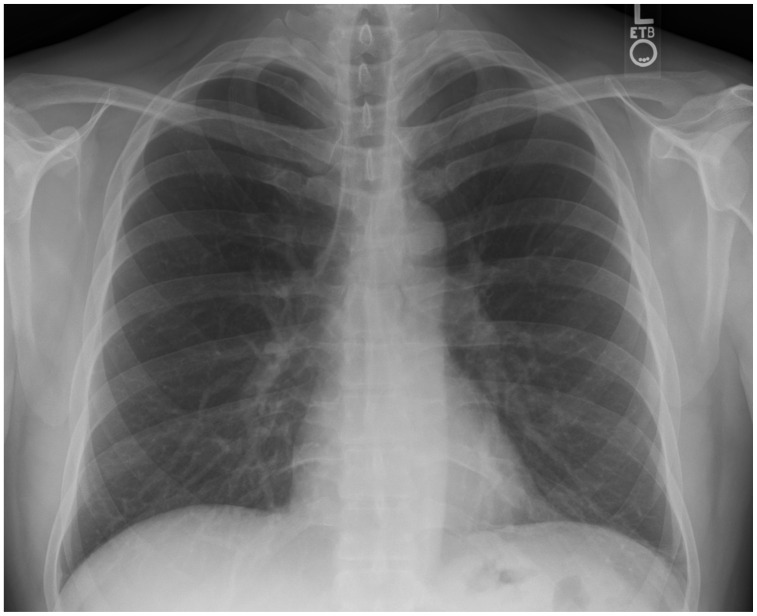


**Table t11-jetem-5-2-s55:** SIMULATION EVENTS TABLE:

Minute (state)	Participant action/trigger	Patient status (simulator response) & operator prompts	Monitor display (vital signs)
0:00 (Baseline)	Patient moved into bed in the emergency department.	Participants should begin by placing the patient on a monitor, obtaining history from wife, and initiating a physical exam.	T 101.3 HR 107 BP 140/84 RR 20 O_2_sat 100% RA
04:00	IV placed, labs drawn. Participant should perform a thorough physical exam. Peripheral smear, labs, UA, and CXR should be ordered.	If the team administers an IV fluid bolus, fever and heart rate will improve. If not, tachycardia and respiratory rate worsen. If the team does not order a peripheral smear within 2 minutes of seeing the CBC results, the lab technician will call and ask if this is something they would like to order.	IV fluid given: T 99.8 HR 94 BP 140/84 RR 20 O_2_sat 100% RA IVF not given T 102.5 HR 120 BP 140/84 RR 24 O_2_sat 100% RA
10:00	Team should suspect blast crisis after reviewing peripheral smear. Broad spectrum antibiotics should be given.	(A) If team performs an LP, patient will complain of low back pain and state “something is wrong with my legs.” Patient will now have 2/5 strength in bilateral lower extremity. The wife will insist that the participants tell her why his legs are now weak. (B) If the hospitalist/intensivist is contacted prior to talking to hematology, they will ask the team to do so given the patient’s history of CML.	IV fluid given: T 99.8 HR 94 BP 140/84 RR 20 O_2_sat 100% RA IVF not given T 101.3 HR 120 BP 140/84 RR 24 O_2_sat 100% RA

**Table t12-jetem-5-2-s55:** 

**Critical Actions:** Obtain a point-of-care glucose prior to obtaining bloodwork results Obtain a peripheral smear Administer appropriate CNS infection treatment without performing LP Emergently consult hematology Admit patient to ICU level of care	0:00

**Table t13-jetem-5-2-s55:** 

	Milestone	Did not achieve level 1	Level 1	Level 2	Level 3
1	**Emergency Stabilization (PC1)**	□ Did not achieve Level 1	□ Recognizes abnormal vital signs	□ Recognizes an unstable patient, requiring intervention Performs primary assessment Discerns data to formulate a diagnostic impression/plan	□ Manages and prioritizes critical actions in a critically ill patient Reassesses after implementing a stabilizing intervention
2	**Performance of focused history and physical (PC2)**	□ Did not achieve Level 1	□ Performs a reliable, comprehensive history and physical exam	□ Performs and communicates a focused history and physical exam based on chief complaint and urgent issues	□ Prioritizes essential components of history and physical exam given dynamic circumstances
3	**Diagnostic studies (PC3)**	□ Did not achieve Level 1	□ Determines the necessity of diagnostic studies	□ Orders appropriate diagnostic studies. Performs appropriate bedside diagnostic studies/procedures	□ Prioritizes essential testing Interprets results of diagnostic studies Reviews risks, benefits, contraindications, and alternatives to a diagnostic study or procedure
4	**Diagnosis (PC4)**	□ Did not achieve Level 1	□ Considers a list of potential diagnoses	□ Considers an appropriate list of potential diagnosis May or may not make correct diagnosis	□ Makes the appropriate diagnosis Considers other potential diagnoses, avoiding premature closure
5	**Pharmacotherapy (PC5)**	□ Did not achieve Level 1	□ Asks patient for drug allergies	□ Selects an medication for therapeutic intervention, consider potential adverse effects	□ Selects the most appropriate medication and understands mechanism of action, effect, and potential side effects Considers and recognizes drug-drug interactions
6	**Observation and reassessment (PC6)**	□ Did not achieve Level 1	□ Reevaluates patient at least one time during case	□ Reevaluates patient after most therapeutic interventions	□ Consistently evaluates the effectiveness of therapies at appropriate intervals
7	**Disposition (PC7)**	□ Did not achieve Level 1	□ Appropriately selects whether to admit or discharge the patient	□ Appropriately selects whether to admit or discharge Involves the expertise of some of the appropriate specialists	□ Educates the patient appropriately about their disposition Assigns patient to an appropriate level of care (ICU/Tele/Floor) Involves expertise of all appropriate specialists
9	**General Approach to Procedures (PC9)**	□ Did not achieve Level 1	□ Identifies pertinent anatomy and physiology for a procedure Uses appropriate Universal Precautions	□ Obtains informed consent Knows indications, contraindications, anatomic landmarks, equipment, anesthetic and procedural technique, and potential complications for common ED procedures	□ Determines a back-up strategy if initial attempts are unsuccessful Correctly interprets results of diagnostic procedure
20	**Professional Values (PROF1)**	□ Did not achieve Level 1	□ Demonstrates caring, honest behavior	□ Exhibits compassion, respect, sensitivity and responsiveness	□ Develops alternative care plans when patients’ personal beliefs and decisions preclude standard care
22	**Patient centered communication (ICS1)**	□ Did not achieve level 1	□ Establishes rapport and demonstrates empathy to patient (and family) Listens effectively	□ Elicits patient’s reason for seeking health care	□ Manages patient expectations in a manner that minimizes potential for stress, conflict, and misunderstanding. Effectively communicates with vulnerable populations, (at risk patients and families)
23	**Team management (ICS2)**	□ Did not achieve level 1	□ Recognizes other members of the patient care team during case (nurse, techs)	□ Communicates pertinent information to other healthcare colleagues	□ Communicates a clear, succinct, and appropriate handoff with specialists and other colleagues Communicates effectively with ancillary staff

## INSTRUCTOR MATERIALS

**Case Title:** Blast Crisis

**Case Description & Diagnosis (short synopsis):** Patient is a 64-year-old male who is brought into the emergency department (ED) by his wife for a headache. He is altered, so additional history is obtained from his wife. He also has been having fevers, vision changes, and confusion. Goals include early recognition of blast crisis and evaluation of other causes of altered mental status in the undifferentiated febrile patient. Participants should obtain a peripheral smear, bloodwork, a chest x-ray, and urinalysis. Once the diagnosis of blast crisis is made, hematology should be immediately consulted for emergent assessment for TKI therapy and leukapheresis. In the meantime, the patient should be placed on the appropriate antibiotics for possible confounding or concurrent infectious etiologies. The patient should be admitted to the intensive care unit (ICU).

**Equipment or Props Needed:**

High fidelity pediatric simulation mannequin

Angiocaths for peripheral intravenous access = 18g, 20g, 22g

Cardiac monitor

Pulse oximetry

IV pole

Normal saline (1L ×2)

Lactated Ringer’s (1L ×2)

Simulated medications with labeling: ceftriaxone, vancomycin, acyclovir, ampicillin

**Confederates needed:**

Primary nurse

Faculty may call in from control room as hematologist and intensivist

**Stimulus Inventory:**

[Table t2-jetem-5-2-s55]

**Background and brief information:** Patient is a 64-year-old male who is brought in by his wife for a day of confusion.

**Initial presentation:** Per his wife, he has been acting confused all day today. He seems confused about where he is and what year it is. He has been sleepier for the past two days and also complains of headache, general pains, and chills. His wife took his temperature today and noted a fever of 101°F. He was recently diagnosed with CML, but treatment has not yet been initiated.

**How the scenario unfolds:** Patient is a 64-year-old male who is brought into the emergency department (ED) by his wife for a headache. He is altered, so additional history is obtained from his wife. He also has been having fevers, vision changes, and confusion. Goals include early recognition of blast crisis and evaluation of other causes of alerted mental status in the undifferentiated febrile patient. Participants should obtain a peripheral smear, bloodwork, a chest x-ray, and urinalysis. Once patient receives an antipyretic and an IV fluid bolus, fever and heart rate will improve. If participants proceed with a lumbar puncture despite thrombocytopenia, patient will report back pain and leg weakness. Once the diagnosis of blast crisis is made, hematology should be immediately consulted for emergent assessment for TKI therapy and leukapheresis. In the meantime, the patient should be placed on the appropriate antibiotics for possible confounding or concurrent infectious etiologies. The patient should be admitted to the intensive care unit (ICU).

**Critical actions:**

1. Obtain a point-of-care glucose prior to obtaining bloodwork results
2. Obtain a peripheral smear
3. Administer appropriate central nervous system (CNS) infection treatment without performing LP
4. Emergently consult hematology
5. Admit patient to ICU level of care

**Case Title:** Blast Crisis

**Chief Complaint:** Patient is a 64-year-old male who is brought in by his wife for a day of confusion.

[Table t3-jetem-5-2-s55]

**General Appearance:** Lying supine, mildly confused, diaphoretic

**Primary Survey:**

- **Airway:** Intact
- **Breathing:** Mildly tachypneic at rest
- **Circulation:** Tachycardic rate and regular rhythm. 2+ symmetric pulses

**History:**

- **History of present illness:** Patient is a 64-year-old male who is brought in by his wife for a day of confusion. Per his wife, he has been acting confused all day today. He seems confused about where he is and what year it is. He has been sleepier the past 2 days and complains of headache, general pains, and chills. His wife took his temperature today and noted a fever of 101oF. He was recently diagnosed with CML, but treatment has not yet been initiated. If asked, she denies that he has had upper respiratory symptoms, cough, nausea, vomiting, diarrhea, hematuria, or a rash. He has not had any recent falls. No sick contacts or recent travel.
- **Past medical history**: CML, hypertension, type 2 diabetes mellitus
- **Past surgical history:** None
- **Medications:** Metformin, lisinopril
- **Allergies:** None
- **Social history:** Rare alcohol use, no tobacco use, no illicit drugs
- **Family history:** Noncontributory

**Secondary Survey/Physical Examination:**

- **General appearance:** Lying supine, mildly confused, diaphoretic.
- **HEENT:**
  ○ **Head:** within normal limits
  ○ **Eyes:** within normal limits
  ○ **Ears:** within normal limits
  ○ **Nose:** within normal limits
  ○ **Throat:** within normal limits
- **Neck:** within normal limits
- **Heart:** regular and tachycardic, otherwise within normal limits
- **Lungs:** mildly tachypneic at rest, otherwise within normal limits
- **Abdominal/GI:** soft, non-tender, non-distended, palpable spleen 3cm below the costal margin
- **Genitourinary:** within normal limits
- **Rectal:** within normal limits
- **Extremities:** within normal limits
- **Back:** within normal limits
- **Neuro:** Mildly confused to date and location. Spontaneously moves all four extremities. No clonus. Cranial nerves intact. Strength intact and 5/5 in all four extremities. No sensory deficits. 2+ reflexes throughout. No cerebellar deficits. Gait is normal.
- **Skin:** within normal limits
- **Lymph:** within normal limits
- **Psych:** within normal limits

**Results:**

[Table t4-jetem-5-2-s55]

*Peripheral smear*

Qasrawi, A. A schematic showing the appearance of acute myeloblastic leukemia, M0 under microscope. In: Wikimedia Commons. https://commons.wikimedia.org/wiki/File:Schematic_showing_the_appearance_of_acute_myeloblastic_leukemia_M0_under_microscope.jpg Published September 16, 2014. Public Domain.[Fig f1-jetem-5-2-s55]

[Table t5-jetem-5-2-s55]

[Table t6-jetem-5-2-s55]

[Table t7-jetem-5-2-s55]

[Table t8-jetem-5-2-s55]

[Table t9-jetem-5-2-s55]

[Table t10-jetem-5-2-s55]

*ECG*

Heilman, J. Sinus tachycardia as seen on ECG. In: Wikimedia Commons. https://commons.wikimedia.org/wiki/File:Sinustachy.JPG. Published June 15, 2012. CC BY-SA 3.0.[Fig f2-jetem-5-2-s55]

*Computed tomography Head*

Ciscel, A. Normal CT scan of the head; this slice shows the cerebellum, a small portion of each temporal lobe, the orbits, and the sinuses. In: Wikimedia Commons. https://commons.wikimedia.org/wiki/File:Head_CT_scan.jpg. Published August 12, 2005. CC BY-SA 2.0.[Fig f3-jetem-5-2-s55]

*CXR*

Stillwaterrising. Chest X-ray PA. In: Wikimedia. https://commons.wikimedia.org/wiki/File:Chest_Xray_PA_3-8-2010.png. Published March 8, 20190. Public Domain.[Fig f4-jetem-5-2-s55]

## OPERATOR MATERIALS

[Table t11-jetem-5-2-s55]

### Diagnosis

Blast Crisis

### Disposition

Admit to the ICU

## DEBRIEFING AND EVALUATION PEARLS

### Spinal Epidural Abscess

#### Pearls

Chronic myeloid leukemia makes up 15%–20% of adult leukemias. CML is characterized by the BCR-ABL fusion gene and the creation of the Philadelphia chromosome. The natural history of myeloid leukemia is characterized by three phases: chronic, accelerated and blast phase.^3^ The progression of CML to blast crisis has been reduced to 1% to 1.5% per year compared to greater than 20% a year in the pre-imatinib era.^2^ Blast crisis is a life-threatening condition characterized by in the increase in blastic cells resulting in hyperviscosity and relative reduction of the other cell lines. The common laboratory features of blast crisis include high white blood cell and blast counts, as well as decreased hemoglobin and platelet counts.

The World Health Organization defines blast crisis as the presence of one or more of the following findings: 20% or greater peripheral blood or bone marrow blasts, large foci or clusters of blasts on bone marrow biopsy, or the presence of extramedullary blastic infiltrates.^3^ Patients who have progressed to the blast phase may present with fever, poor appetite, night sweats, bone pain and weight loss. Therefore, what used to be a chronic leukemia now presents like an acute leukemia.^4^

Hyperleukocytosis is defined as a WBC above 100,000/μL due to leukemic cell proliferation. This can lead to complications such as leukostasis, tumor lysis syndrome, and disseminated intravascular coagulation.^6^ Leukostasis is a clinical diagnosis as a result from blood hyperviscosity and the formation of WBC plugs in the microvasculature. These plugs subsequently lead to decreased tissue perfusion which may lead to end-organ damage. All the immature precursor cells “crowd out” other cell lines, leading to functional anemia, thrombocytopenia, and neutropenia.

The most common cause of death in blast crisis is an infection due to functional neutropenia, followed by hemorrhage due to functional thrombocytopenia. Leukostasis requires emergent treatment and placement in an intensive care unit for aggressive monitoring.

Patients may present with a variety of symptoms affecting many systems due to leukostasis. Most commonly, patients in blast crisis present with neurologic or respiratory complaints.^6^ They may also develop acute coronary syndromes, limb ischemia, bowel infarction, renal insufficiency, and priapism. Extramedullary blast crisis occurs when leukemic blasts infiltrate areas outside of bone marrow, such as the paravertebral scalp to cause spinal cord compression, leukemic ascites, eyes to cause enucleation, and osteolytic bone lesions.

Patients will require a broad work up to rule out other potential causes of their symptoms. In this case, the patient presented with neurologic complaints secondary to leukostasis. Common neurologic complaints include visual changes, headache, dizziness, tinnitus, gait instability and confusion. Without a thorough work-up, other diagnoses such as stroke, meningitis, and encephalitis cannot be excluded.

Overall, the emergency provider should treat any sign of infection with broad spectrum antibiotics. Fever may be due to leukostasis or concurrent infection. The patient should be adequately fluid resuscitated while work up is ensuing.

According to the NCI’s PDQ cancer information summary about CML: treatment in the blast phase consists of TKIs, chemotherapy, hydroxyurea, and bone marrow transplantation. TKI therapy consists of Imatinib, Dasatinib, and Nilotinib. Two trials involving Imatinib and one trial with Dasatinib showed a hematologic response rate of 42% to 55% and a major cytogenetic response rate of 16% to 25%. Often TKIs may be combined with a chemotherapy agent such as Vincristine and prednisone. Bone marrow transplantation is the only potentially curative therapy for these patients. Bone marrow transplant is more effective in those patients who can be induced into a second chronic phase.^5^

#### Other debriefing Points

As an emergency medicine provider, it is important to know your state laws when obtaining consent from the next of kin. When a patient lacks the capacity to make their own medical decisions, the next of kin should be the one to provide consent. Depending on your state laws and the situation, the next of kin may be a spouse or a child. In the case that a health care power of attorney is designated, this person becomes the appropriate person from whom to obtain consent. When obtaining consent, it is important to discuss the diagnosis, indications, benefits, risks and alternatives, so that an informed decision can be made.^7^

This case can also be used to review the steps, indications and contraindications to performing a lumbar puncture (LP). Contraindications include increased intracranial pressure due to a central nervous system lesion, ongoing anticoagulant therapy, or overlying skin infection. After consent is obtained, the patient should be placed in the lateral decubitus position if an opening pressure is to be obtained. The iliac crests should be palpated and used to guide in locating the L3–L4 and L4–L5 spaces. These are the safest spaces to enter your spinal needle because it is well below the conus medullaris in most patients. The overlying skin should be cleaned with alcohol and a disinfectant such as povidone-iodine. Local anesthesia with lidocaine is infiltrated into the skin and the chosen lumbar intervertebral space. The spinal needle with the stylet in place is then advanced slowly, with the needle slightly angulated towards the head. Advance the needle until CSF flow is achieved for collection.^8^

In this case with marked thrombocytopenia, the patient should be empirically treated for encephalitis. Studies are still lacking to support a clear indication to transfuse platelets prior to LP. Some may use a cut off platelet count of ≤ 50 × 10^9/l.^9^ In conclusion, per a recent Cochrane systemic review, there currently is no evidence from RCTs or non-randomized studies on which to base an assessment of the correct platelet transfusion threshold prior to insertion of a lumbar puncture needle.^10^ Therefore, with lacking studies, it is difficult to create a hard platelet cut-off for when it is safe to perform an LP or to determine when a preprocedural platelet transfusion would be indicated.

#### Other debriefing points

Closed-loop communication amongst team: was it used? Why or why not? Were there any implications of this during case execution?

#### Important history or Information to obtain/consider

When was the patient diagnosed with CML? Are they undergoing treatment? Do they wish to receive treatment for their CML or just symptomatic relief?

#### Important Disposition information to know about your hospital

Does your hospital have a hematologist/oncologist on call?

Does your hospital have the staff and capabilities to perform emergency leukapheresis?

**Wrap Up:** *Brief wrap up lecture (optional), references and/or suggestions for further reading. Please also include any other optional associated content here (worksheets for observing learners, etc).*

## SIMULATION ASSESSMENT

*Blast Crisis*

Learner: _________________________________________

***Assessment Timeline***

This timeline is to help observers assess their learners. It allows observer to make notes on when learners performed various tasks, which can help guide debriefing discussion.

[Table t12-jetem-5-2-s55]

**Critical Actions:**

□ Obtain a point-of-care glucose prior to obtaining bloodwork results
□ Obtain a peripheral smear
□ Administer appropriate CNS infection treatment without performing LP
□ Emergently consult hematology
□ Admit patient to ICU level of care

**Summative and formative comments:**

**Milestones assessment:**

[Table t13-jetem-5-2-s55]
