# Supplementary material for: Blast Crisis
Source: J Educ Teach Emerg Med. 2020 Apr 15;5(2):S55–77. doi: 10.21980/J8W35K (PMC10332564; doi:10.21980/J8W35K)

## Slide 1
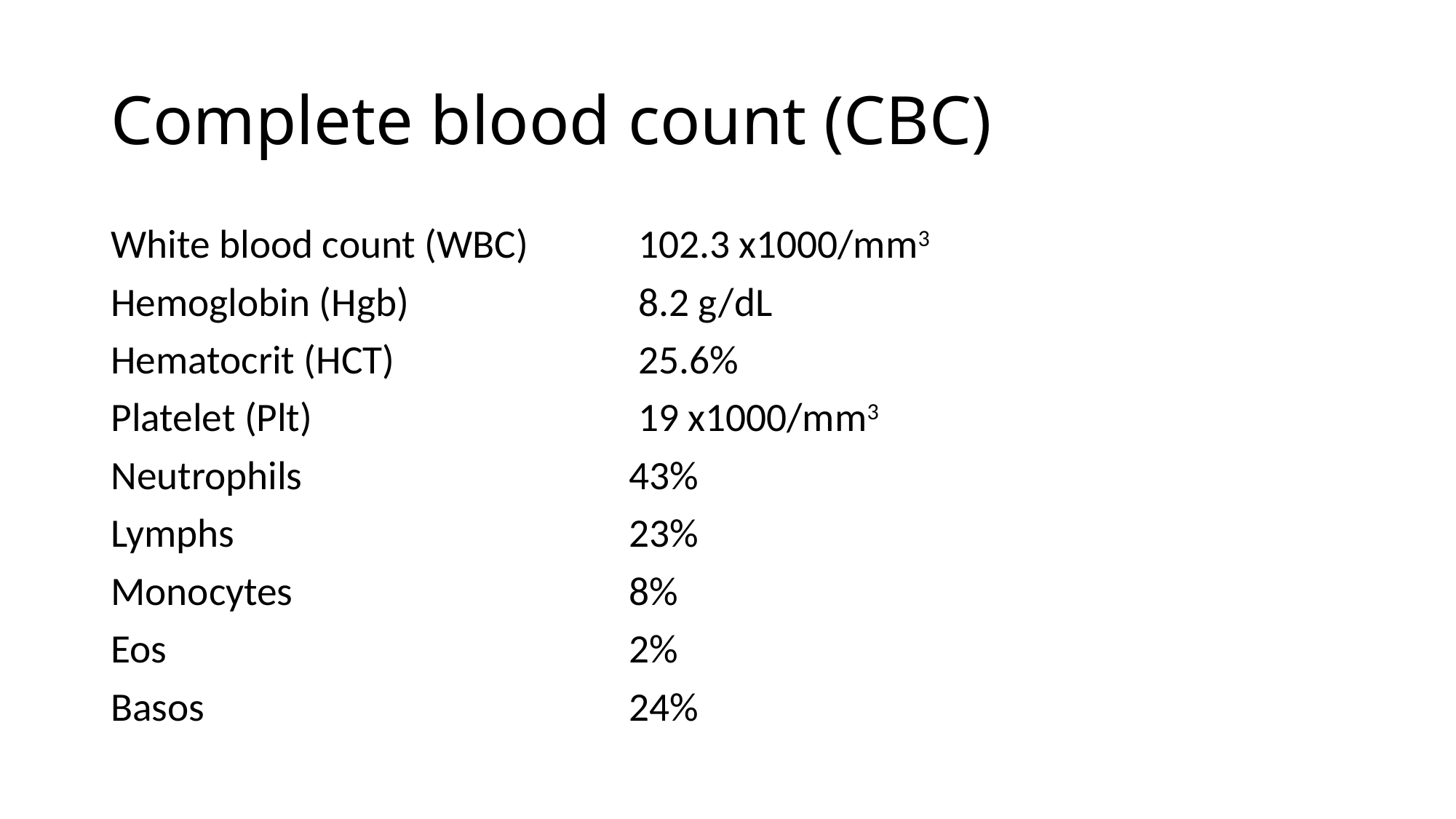

# Complete blood count (CBC)
White blood count (WBC) 	 102.3 x1000/mm3
Hemoglobin (Hgb) 		 8.2 g/dL
Hematocrit (HCT) 		 25.6%
Platelet (Plt) 			 19 x1000/mm3
Neutrophils 				43%
Lymphs 				23%
Monocytes 				8%
Eos 					2%
Basos 					24%

## Slide 2
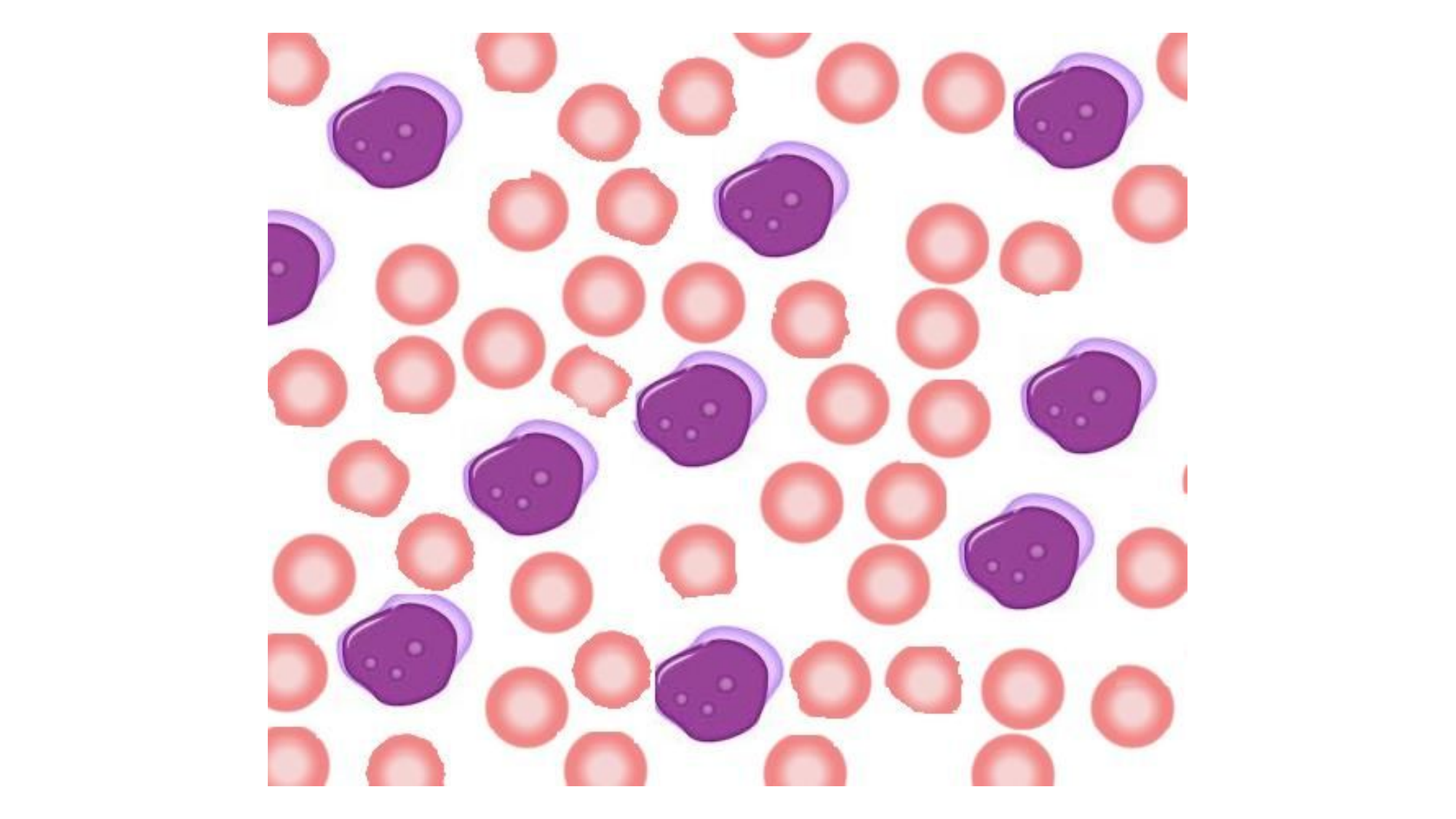

## Slide 3
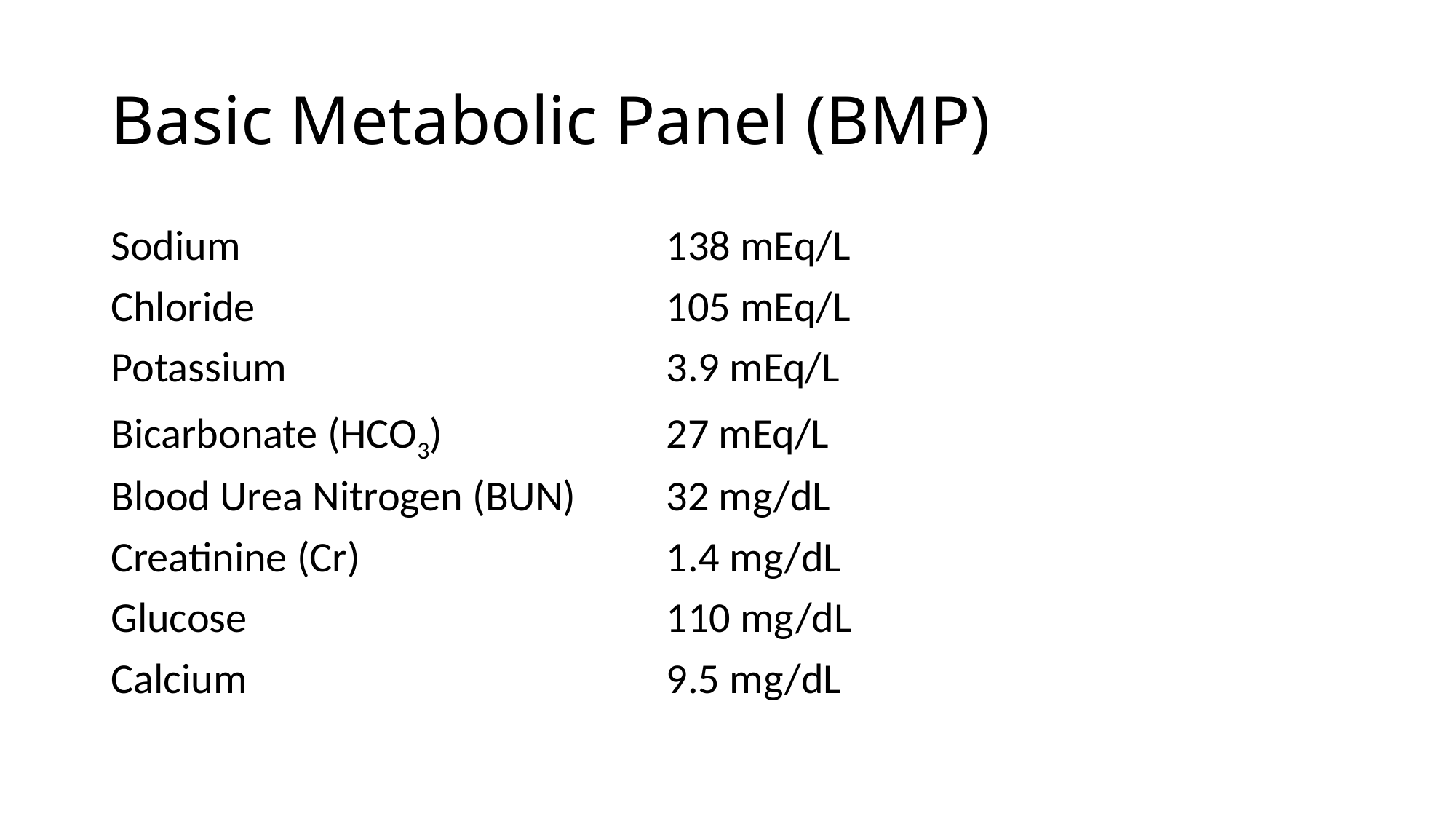

# Basic Metabolic Panel (BMP)
Sodium 				 138 mEq/L
Chloride 				 105 mEq/L
Potassium 				 3.9 mEq/L
Bicarbonate (HCO3) 		 27 mEq/L
Blood Urea Nitrogen (BUN) 	 32 mg/dL
Creatinine (Cr) 			 1.4 mg/dL
Glucose 				 110 mg/dL
Calcium				 9.5 mg/dL

## Slide 4
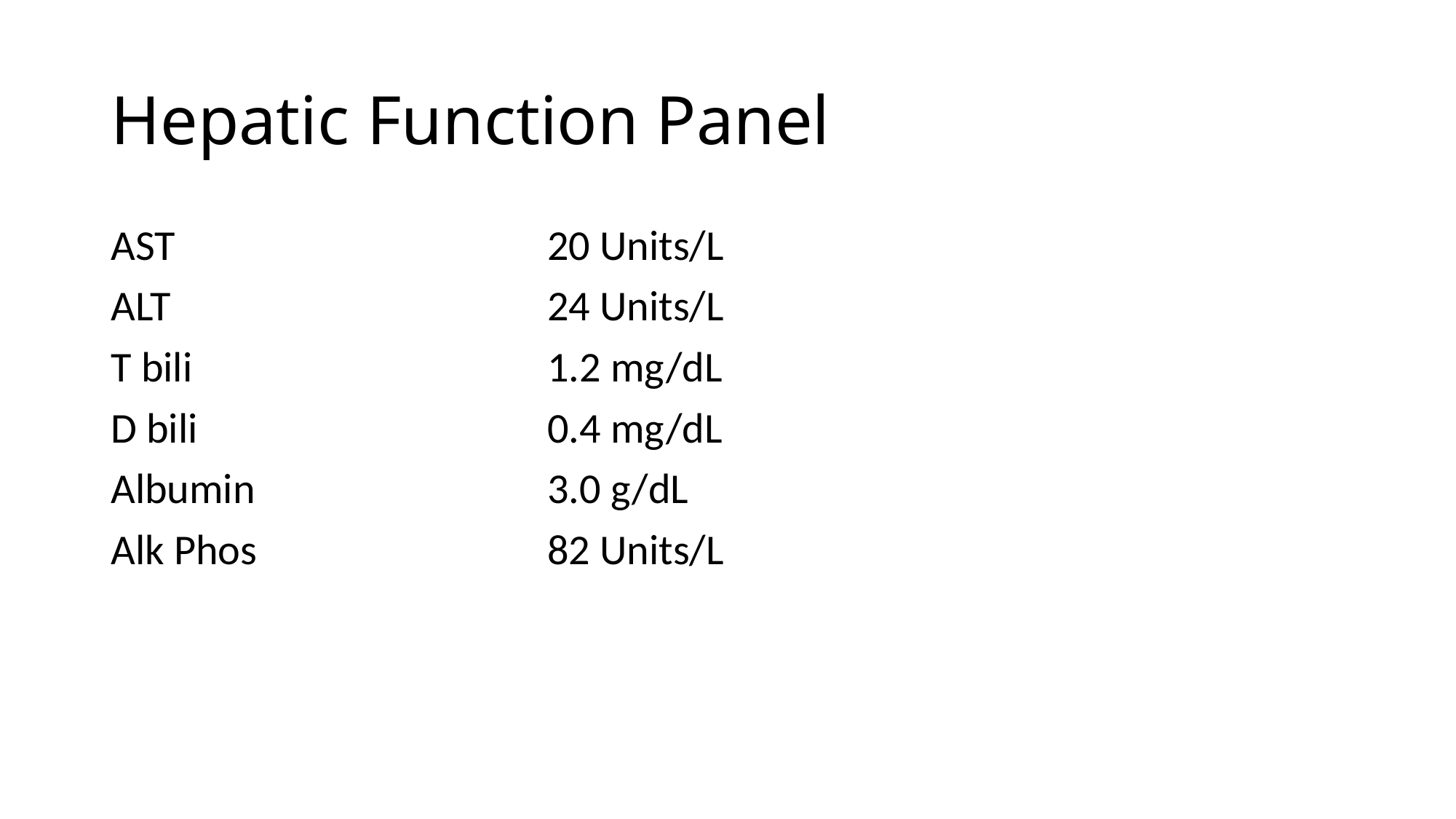

# Hepatic Function Panel
AST 				20 Units/L
ALT 				24 Units/L
T bili 				1.2 mg/dL
D bili 				0.4 mg/dL
Albumin 			3.0 g/dL
Alk Phos 			82 Units/L

## Slide 5
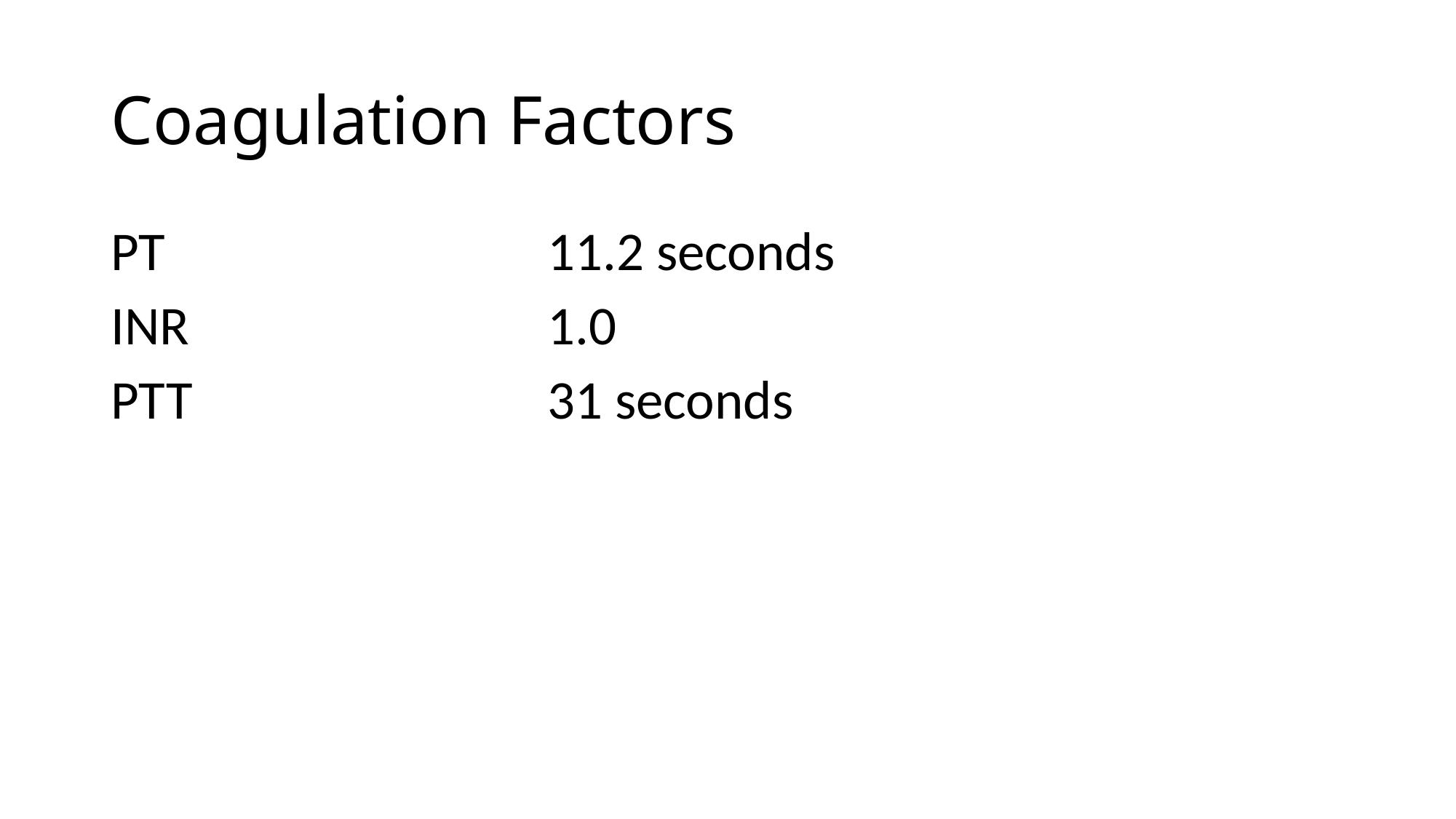

# Coagulation Factors
PT 				11.2 seconds
INR 				1.0
PTT 				31 seconds

## Slide 6
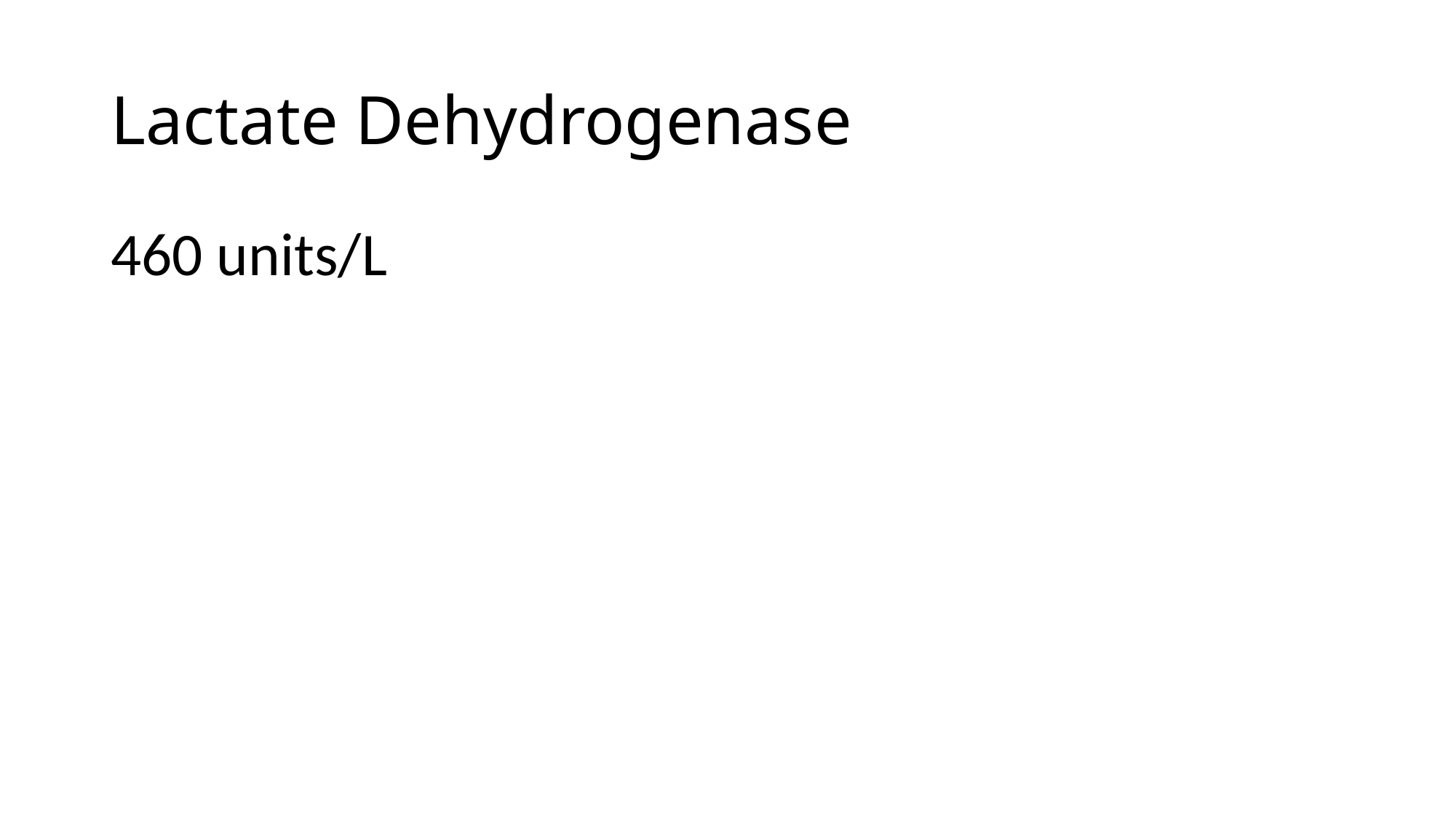

# Lactate Dehydrogenase
460 units/L

## Slide 7
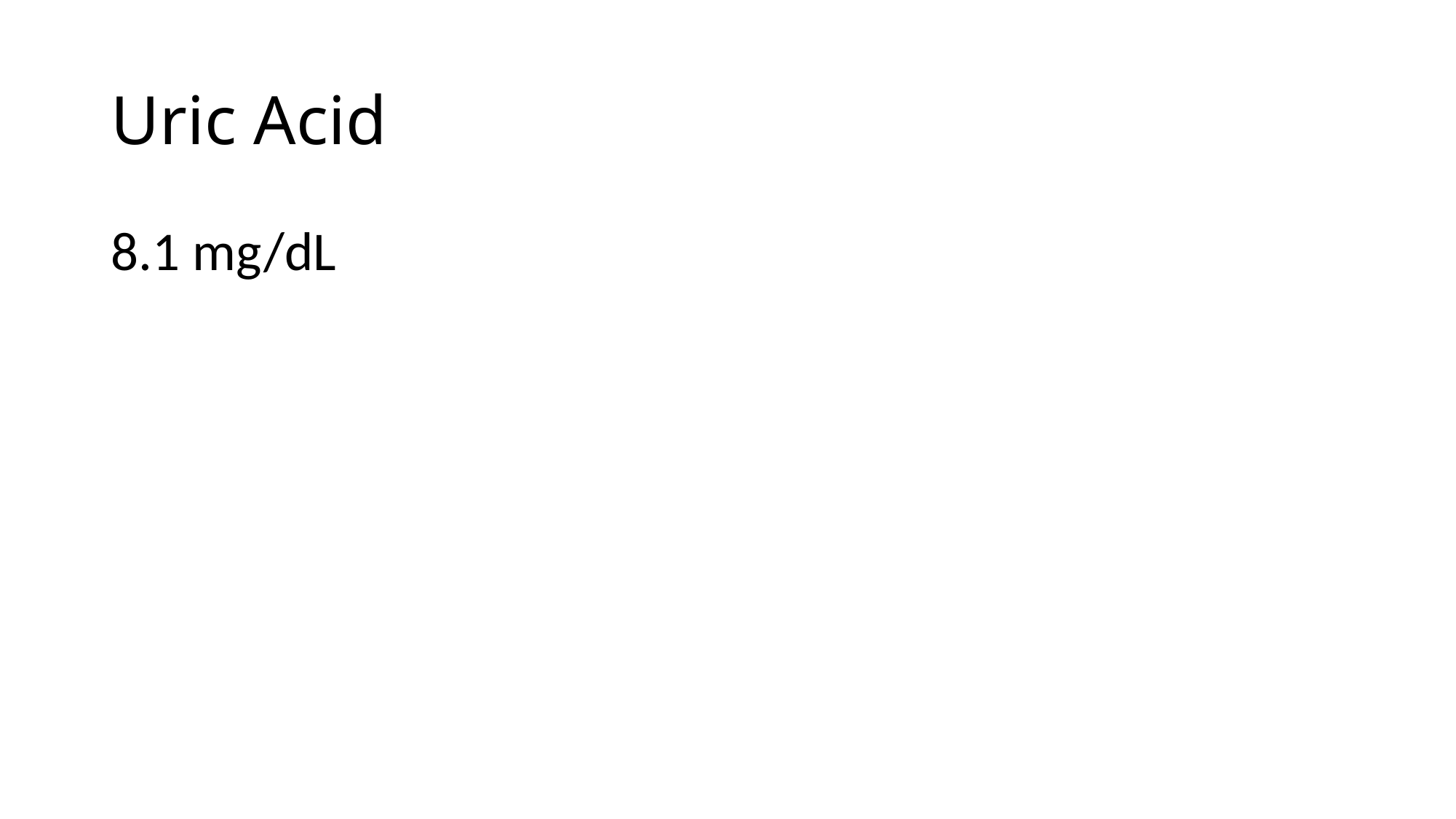

# Uric Acid
8.1 mg/dL

## Slide 8
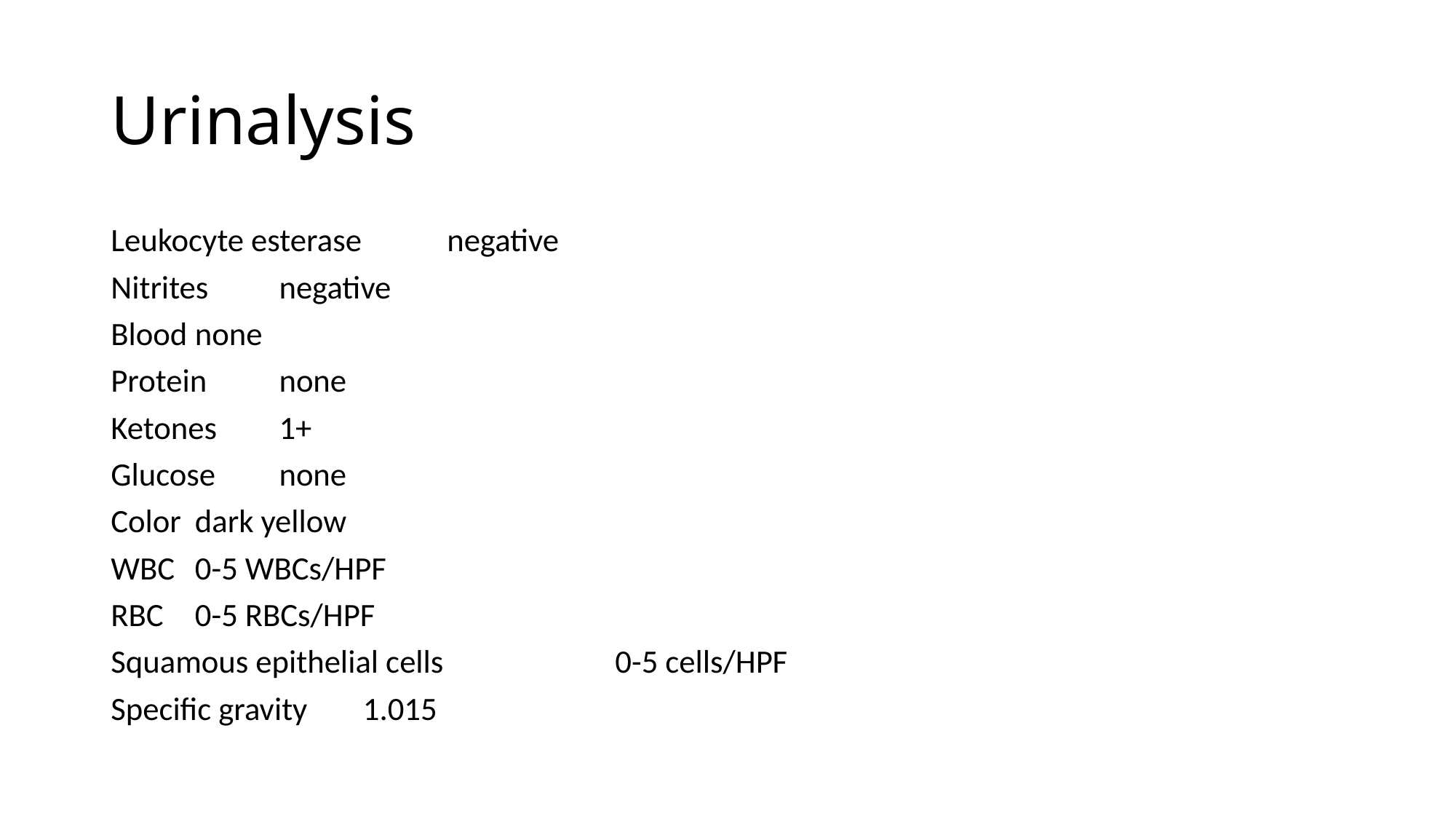

# Urinalysis
Leukocyte esterase 			negative
Nitrites					negative
Blood 					none
Protein					none
Ketones 				1+
Glucose 				none
Color 					dark yellow
WBC 					0-5 WBCs/HPF
RBC 					0-5 RBCs/HPF
Squamous epithelial cells 		0-5 cells/HPF
Specific gravity 			1.015

## Slide 9
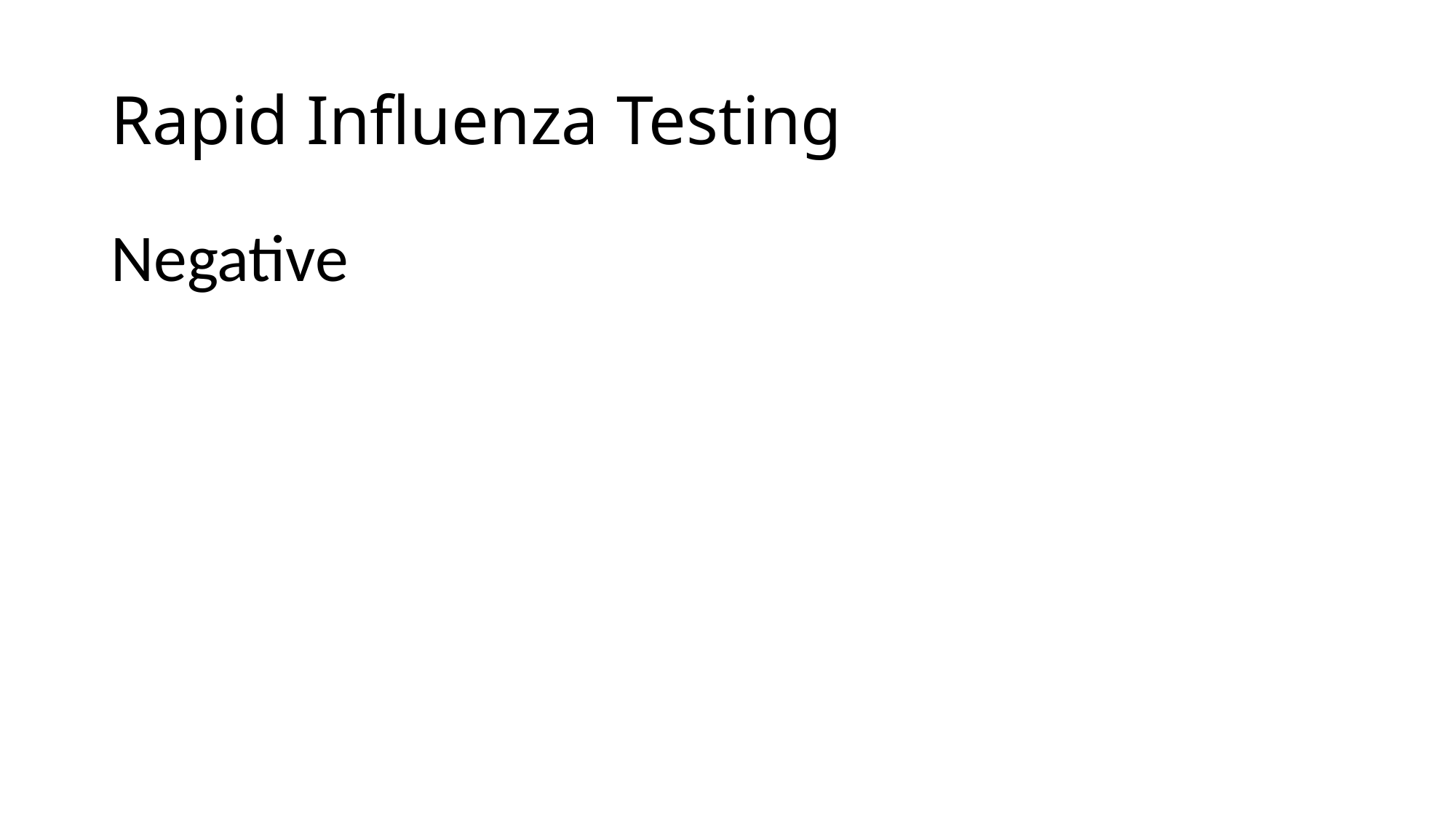

# Rapid Influenza Testing
Negative

## Slide 10
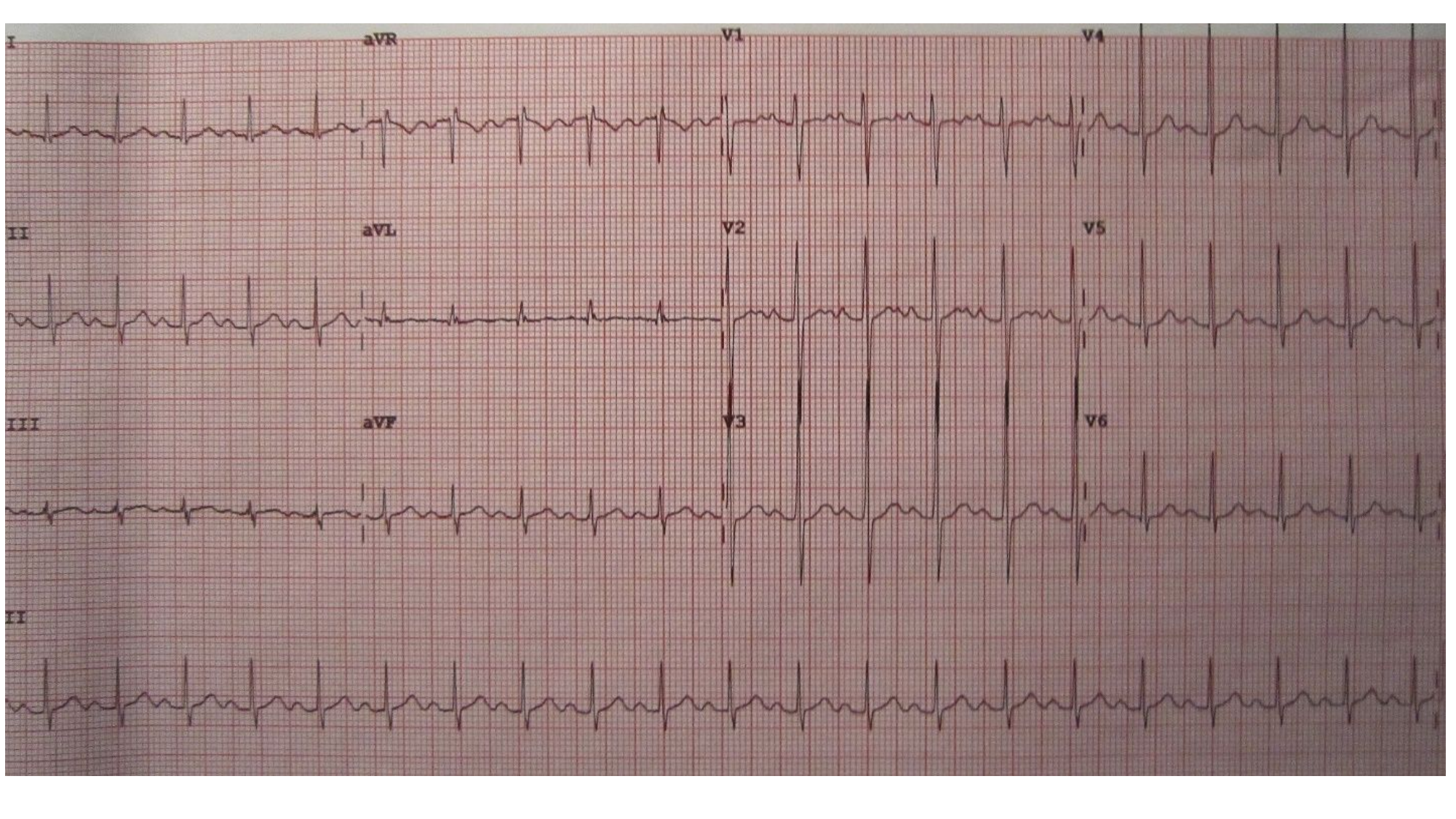

#

## Slide 11
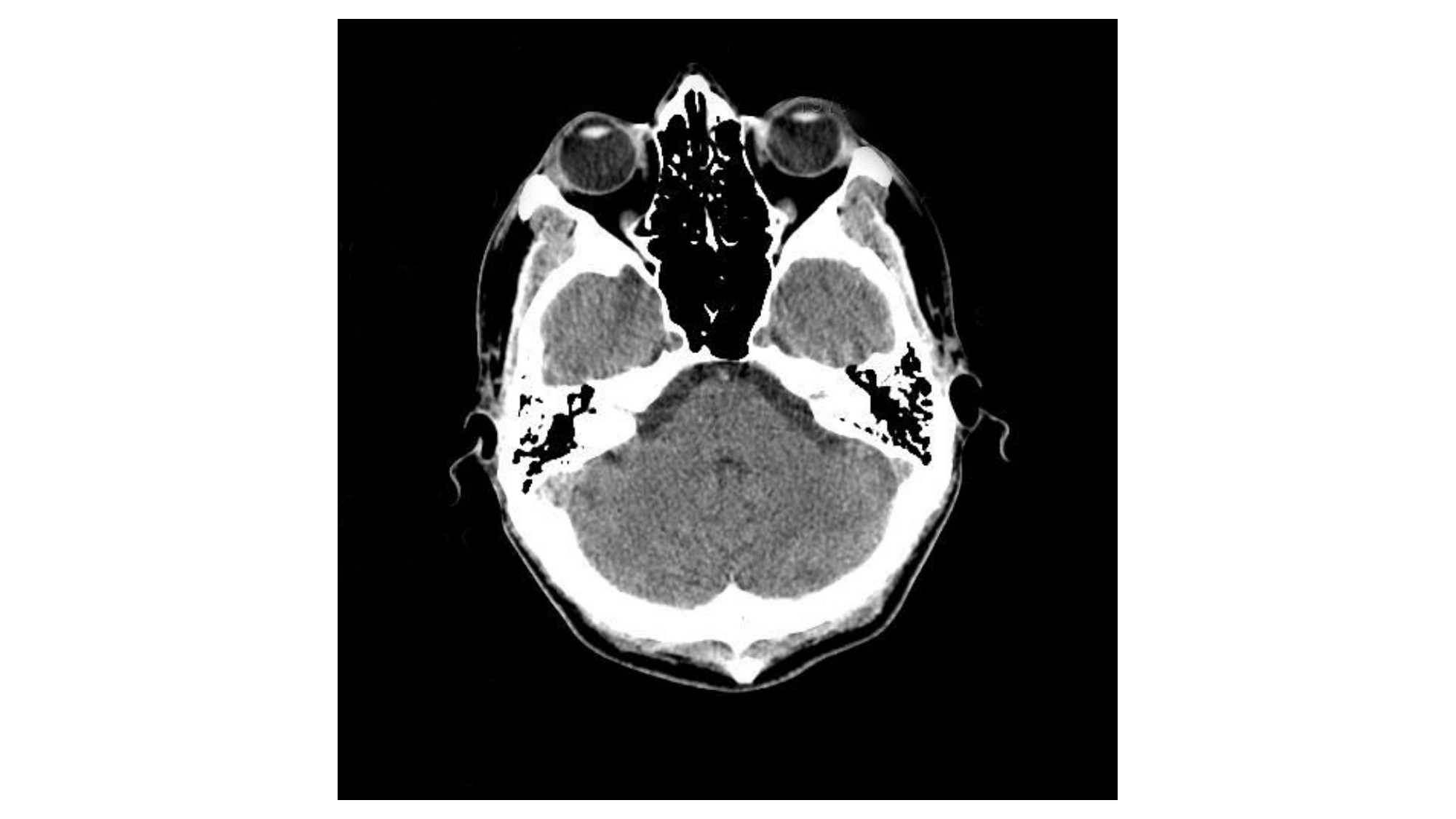

## Slide 12
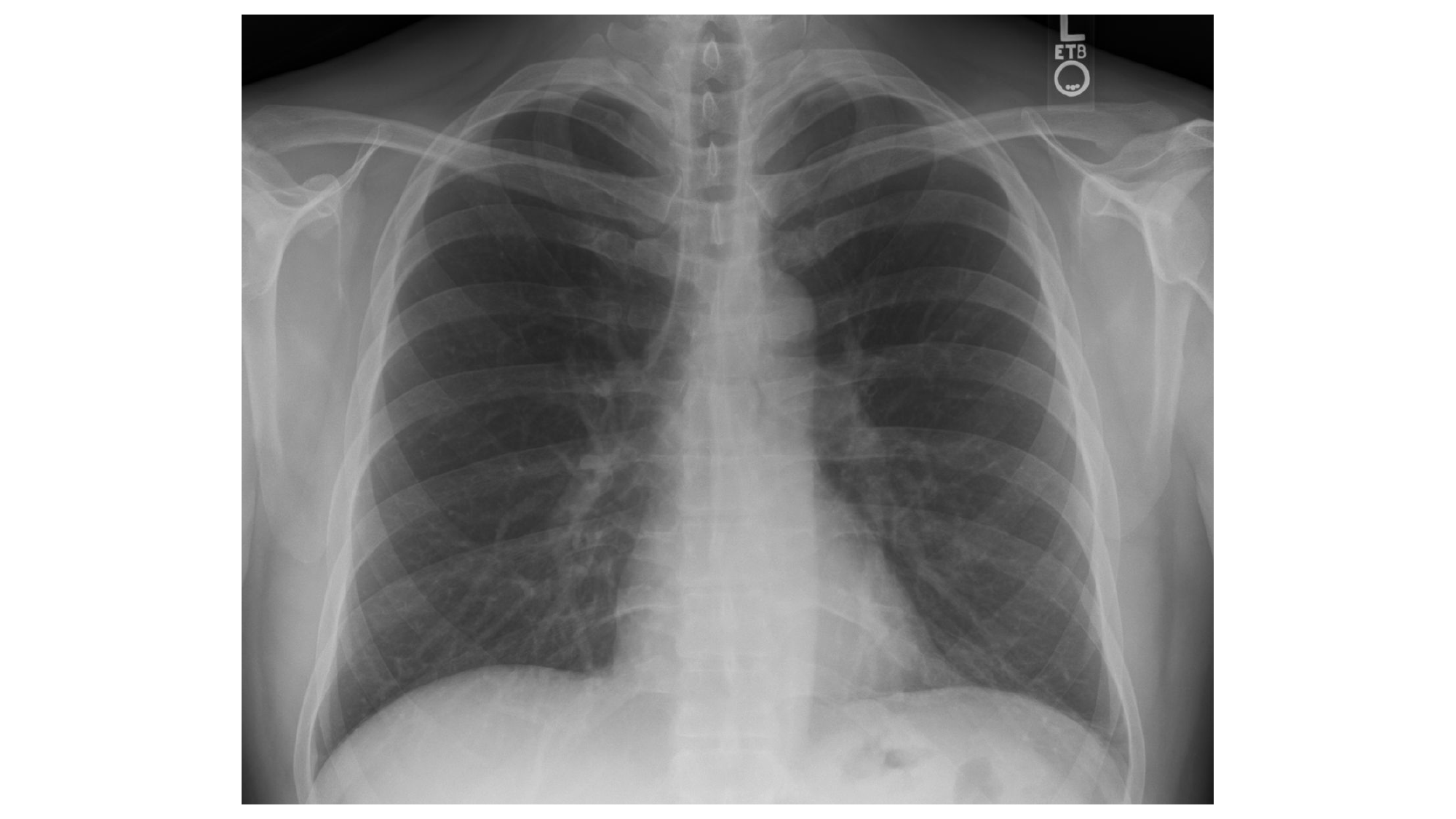

Supplement: Supplementary file 1 [file jetem-5-2-s55-supp1.pptx]
